# The Histone Methyltransferase SUV39H1 Suppresses Embryonal Rhabdomyosarcoma Formation in Zebrafish

**DOI:** 10.1371/journal.pone.0064969

**Published:** 2013-05-21

**Authors:** Colleen E. Albacker, Narie Y. Storer, Erin M. Langdon, Anthony DiBiase, Yi Zhou, David M. Langenau, Leonard I. Zon

**Affiliations:** 1 Stem Cell Program and Division of Hematology/Oncology, Boston Children's Hospital and Dana Farber Cancer Institute, Howard Hughes Medical Institute, Harvard Stem Cell Institute, Harvard Medical School, Boston, Massachusetts, United States of America; 2 Department of Pathology, Massachusetts General Hospital, Harvard Stem Cell Institute, Charlestown, Massachusetts, United States of America; Texas A&M University, United States of America

## Abstract

Epigenetics, or the reversible and heritable marks of gene regulation not including DNA sequence, encompasses chromatin modifications on both the DNA and histones and is as important as the DNA sequence itself. Chromatin-modifying factors are playing an increasingly important role in tumorigenesis, particularly among pediatric rhabdomyosarcomas (RMS), revealing potential novel therapeutic targets. We performed an overexpression screen of chromatin-modifying factors in a KRAS^G12D^-driven zebrafish model for RMS. Here, we describe the identification of a histone H3 lysine 9 histone methyltransferase, SUV39H1, as a suppressor of embryonal RMS formation in zebrafish. This suppression is specific to the histone methyltransferase activity of SUV39H1, as point mutations in the SET domain lacked the effect. SUV39H1-overexpressing and control tumors have a similar proliferation rate, muscle differentiation state, and tumor growth rate. Strikingly, SUV39H1-overexpressing fish initiate fewer tumors, which results in the observed suppressive phenotype. We demonstrate that the delayed tumor onset occurs between 5 and 7 days post fertilization. Gene expression profiling at these stages revealed that in the context of KRAS^G12D^ overexpression, SUV39H1 may suppress cell cycle progression. Our studies provide evidence for the role of SUV39H1 as a tumor suppressor.

## Introduction

Rhabdomyosarcoma (RMS) is a pediatric cancer representing more than half of all soft tissue sarcomas in children. 350 new cases arise each year in the United States, with two-thirds of those occurring in children under the age of ten [1]–[3]. RMS tumors are generally sporadic and tend to occur more frequently in boys than girls [1]. The overall survival rate when including non-metastatic cases is currently nearly 80%, as compared to only 25% in the 1970s [4]. This is likely due to advancements in molecular biology techniques that allow for improved diagnosis and imaging, leading to tailored therapies. However, in about 20% of cases, the disease is metastatic at presentation, and even with aggressive treatments, five-year survival rates hover around 20%, suggesting there is still much to learn about the biology of RMS [1].

There are two main histological subtypes of RMS. The embryonal RMS (ERMS) subtype consists of 80% of RMS cases, is mainly in the pediatric population, and typically has a better prognosis. ERMS is characterized by mutations or dysregulation of the RAS pathway and loss of heterozygosity at BWR1A [5]–[10]. A transgenic model of ERMS has previously been developed in the zebrafish, accomplished by driving expression of oncogenic human KRAS^G12D^ with the *rag2* promoter, which was shown to drive expression in mononuclear muscle satellite cells. The fish begin developing tumors as larvae and express the traditional clinical markers of ERMS, including *myogenin*, *myod*, and *desmin*, equivalent to the pediatric patients [5]. The alveolar subtype (ARMS) is more likely to occur in adolescents, be metastatic, and have a poorer prognosis. ARMS is caused by a chromosomal translocation between either Pax3 or Pax7 and forkhead transcription factors [1], [8], [11].

Though little is known about the role of chromatin-modifying factors in RMS, several studies have implicated components of the Polycomb Group and SWI/SNF chromatin remodeling complex. Polycomb Group member YY1 was found to be upregulated in RMS cell lines and primary tumors, thus leading to recruitment of EZH2 and HDAC1 to miR-29, silencing this microRNA, and thereby preventing muscle differentiation and facilitating tumor development [12]. Human SNF5 homolog, BAF47, was noted to be mutated or deleted in 25% of primary tumors and 10% of RMS cell lines analyzed [13], [14]. Upon treatment with 12-O-Tetradecanoylphorbol-13-acetate (TPA), the ERMS cell line RD differentiates through a mechanism involving PCAF and the BRG1 subunit of the SWI/SNF complex being sequentially recruited to the *myogenin* promoter, representing a novel therapeutic strategy to induce tumors differentiation *in vivo* [15]. Chromatin factors may also represent useful diagnostic markers or novel drug targets in RMS. For instance, the histone demethylase LSD1 was shown to have high expression levels in malignant sarcomas, including ARMS (2/2) and ERMS (6/7) tumors; it may prove useful as a diagnostic marker or a novel drug target [16], [17]. Since the regulation of chromatin structure can play a determinative role in the formation and behavior of cancers of the muscle, it is likely that many more chromatin factors participate in RMS but remain to be discovered.

Here, we used an injection-based screening approach in zebrafish to interrogate the role of nineteen chromatin-modifying factors in RMS formation. We identified histone methyltransferase SUV39H1 as a strong suppressor of RMS formation, and this effect was dependent on an active SET domain. While SUV39H1 did not impact overall tumor characteristics when compared to control tumors, including histological and gene expression analyses, studies of tumor initiation using a fluorescent monitoring system demonstrated that SUV39H1 acts between 5 and 7 days post fertilization (dpf) to delay the onset of tumor formation. Gene expression studies also demonstrate a potential cell cycle regulation defect in SUV39H1 injected embryos. This data suggests a model in which altered cell cycle regulation caused by SUV39H1 overexpression is responsible for the decrease in RMS tumor initiation.

## Methods

### Zebrafish

Zebrafish were maintained and developmentally staged as previously described according to IACUC guidelines [18]. The Animal Care and Use Committee, Children's Hospital Boston approved all animal protocols.

### Vectors and cloning

The *rag2*-hKRAS^G12D^ and *rag2*-GFP vectors were previously described [5]. The *mylz2*-GFP and mCherry vectors were previously described [19]. To create the rag2 destination vector, the *rag2*-hKRAS^G12D^ vector was digested with BamHI and HindIII, blunt-ended with Klenow, incubated with Shrimp Alkaline Phosphatase and purified; the *rag2* destination vector for Gateway cloning was then constructed from the blunt-ended vector using the Gateway Vector Conversion System (Invitrogen, Life Technologies, Grand Island, NY). *rag2*-hSUV39H1^H324K^ and *rag2*-hSUV39H1^C326A^ vectors were obtained from C. Ceol [20]. *rag2*-chromatin factor expression vectors were generated by Gateway recombination using human, full-length open reading frames from the Ultimate ORF Clone collection (Invitrogen, Life Technologies, Grand Island, NY). A pENTR-mPAX7 vector was obtained from Open Biosystems (Thermo Fisher Scientific, Huntsville, AL) and was put behind the *rag2* promoter through Gateway recombination. All constructs were sequence verified.

### Microinjection and tumor scoring

The *rag2-*hKRAS^G12D^, *rag2-*chromatin factor, and all fluorescent protein/SUV39H1-containing vectors were linearized with XhoI, purified, and diluted in 0.5×TE+0.1 M KCl. For co-injection of three transgenes, each was diluted to 40 ng/uL, and for co-injection of four transgenes, each was diluted to 30 ng/uL. One nL of the vector dilutions was microinjected into the nucleus of one-cell stage AB strain zebrafish embryos. For the screen, fish were scored for visible tumor formation every 2–4 days commencing at 12 dpf. For younger larvae, fish were scored for tumor formation by presence of fluorescence every 2–3 days commencing at 6 dpf. For the tumor growth analysis, fluorescent photos of each fish were taken at the same zoom and magnification, and photos were analyzed for number of fluorescent pixels on ImageJ.

### Identification of known and putative chromatin modifying factors

Human chromatin modifying factors were identified using CREMOFAC, SMART domain, CDD, and Pfam databases.

### Gene set enrichment analysis

GSEA on published human microarray data sets was performed as described previously [5], [21].

### Statistical analysis

Tumor-free survival over time is graphically represented as a Kaplan-Meier estimate of survival using GraphPad Prism (La Jolla, CA). The log-rank test was used to compare survival of experimental and control groups.

### Microarray analysis

RNA was isolated at 5 and 7 dpf from approximately twenty sibling embryos per sample, with three biological replicates, for each of the *rag2*-mCherry and *rag2*-hSUV39H1 types with the RNeasy Mini Kit (Qiagen, Valencia, CA) using the animal tissue protocol and subsequently treated with DNase I. cDNA was prepared and hybridized to zebrafish Affymetrix arrays according to manufacturer's instructions. Genes differentially regulated between the tumor types were identified (>2-fold change, p<0.05).

### EdU incorporation

Tumor-bearing fish at 28 to 30 dpf were injected intraperitoneally with 10 ul of 2.5 mg/ml EdU per 0.25 g body weight. After 24 hours, fish were euthanized and frozen in Optimal Cutting Temperature (OCT) medium at −80°C overnight. 12 um cryostat sections were prepared for each tumor, and EdU labeling was performed using the Click-iT EdU Alexa Fluor 594 Imaging Kit (Invitrogen, Life Technologies, Grand Island, NY). Labeled sections were imaged at 400× magnification using a compound fluorescent microscope, and the number of EdU-positive and DAPI-positive nuclei were counted in three separate 1.37×10^4^ um^2^ fields per tumor. The ratios of EdU-positive to DAPI-positive nuclei in the three fields were averaged to calculate an EdU/DAPI ratio for each tumor.

### Histopathology

Fish were euthanized and fixed in 4% paraformaldehyde overnight at 4°C and then decalcified in 0.5 M EDTA, pH8. Paraffin embedding, sectioning, and H&E staining were performed according to standard techniques by the Brigham & Women's Pathology Core.

### TUNEL staining

Staining for TUNEL was completed on sections of 5 and 7 dpf highly mosaic larvae injected with *rag2*-hKRAS^G12D^, *rag2*-hSUV39H1 or mCherry, and *mylz2*-GFP. Larvae were fixed in 4% paraformaldehyde (PFA) overnight, bleached to remove melanocytes in a 3% H_2_O_2_/0.5% KOH solution for 45 minutes, then fixed again overnight in 4% PFA. Following paraffin embedding and sectioning, larvae sections were then stained according to manufacturer's instructions using the ApopTag® Plus Peroxidase *In Situ* Apoptosis Kit (Millipore, Billerica, MA). Sections were imaged at 20×, and TUNEL-positive cells were counted in two separate musculature fields in five larvae of each injected type.

### Quantitative RT-PCR

For analysis of larvae, RNA was isolated at 7dpf from 12 sibling larvae per sample, 3 samples total, for each of the *rag2-*mCherry and *rag2-*hSUV39H1 types with the RNeasy Mini Kit (Qiagen, Valencia, CA) and treated with DNaseI. For tumor analysis, RNA was isolated at ∼30dpf from five tumors (one tumor per sample) for each of the *rag2-*mCherry and *rag2-*hSUV39H1 types using TRIzol reagent (Invitrogen), treated with DNase I, and purified using the RNeasy Mini Kit (Qiagen). cDNA synthesis was performed from equal quantities of RNA using SuperScript III First-Strand Synthesis SuperMix for qRT-PCR (Invitrogen), and quantitative RT-PCR was performed using SYBR GreenER qPCR SuperMix for iCycler (Invitrogen) on a BioRad C1000 Thermal Cycler. Primers used for QPCR are included in Table S1. For each sample, relative gene expression was calculated from experimental triplicates using the 2^−ΔΔCT^ method, with normalization to *EF1-alpha* transcript levels within each sample. Normalized relative gene expression was then averaged across samples for each group, and gene transcript expression levels between *rag2-*mCherry and *rag2-*hSUV39H1 types were compared using the student's t-test.

## Results

### An *in vivo* overexpression screen in zebrafish to identify modifiers of embryonal rhabdomyosarcoma

**Figure 1:**
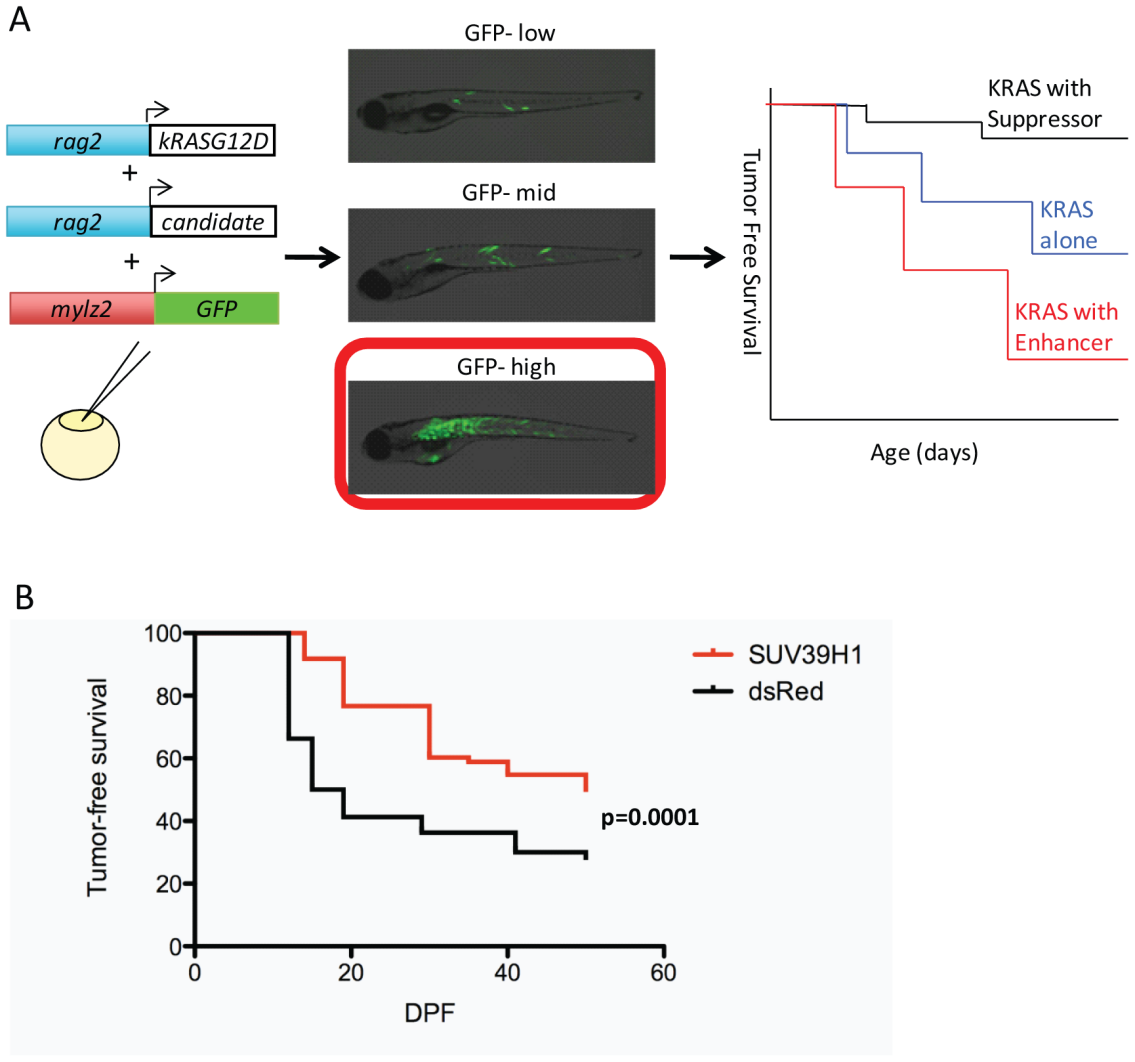
An overexpression screen reveals SUV39H1 as a suppressor of rhabdomyosarcoma formation in zebrafish. (A) Three linearized DNA constructs were injected into one-cell stage embryos, *rag2*-hKRAS^G12D^, *rag2*-chromatin factor, and *mylz2*-GFP. At 2 dpf, embryos were scored for GFP mosaicism; only those that were GFP-high were kept for tumor evaluation. Tumor-free survival curves were then constructed for days 12–50 of life, looking for enhancers or suppressors of tumor formation compared to control injected zebrafish with *rag2*-hKRAS^G12D^ without a modifier gene. (B) SUV39H1 significantly suppressed RMS formation, compared to a control curve where the modifier gene was dsRed (SUV39H1 n = 73, dsRed n = 80, p = 0.0001).

To identify chromatin-modifying factors that act as modifiers of RMS, we utilized a previously characterized model of ERMS in the zebrafish [5]. This model was based on a microinjection strategy amenable to co-injection of different factors with their expression driven by the *rag2* promoter. Injection of a *rag2*-hKRAS^G12D^ construct drives tumor formation; additional genes on separate, linearized plasmids are also driven by *rag2* and co-integrate with and are co-expressed in the *rag2*-hKRAS^G12D^ tumors [5], [22]. Therefore, we developed a strategy to identify suppressors or enhancers of RMS formation when the candidate gene, driven by *rag2*, was co-injected with *rag2*-hKRAS^G12D^. To control for injection variability, a third construct, *mylz2 (myosin light polypeptide 2)*-GFP, was co-injected. This allowed the microinjected embryos to be separated into categories of *mylz2-*GFP-low, middle, and high mosaicism at 2 dpf. The level of GFP mosaicism directly correlated with successful microinjection of the hKRAS^G12D^ and therefore tumor formation (data not shown). Those with high GFP mosaicism were selected and visually analyzed for tumor formation every 3 days from 12 to 50 dpf (Figure 1A).

We developed a list of chromatin factors to test for enhancement or suppression of RMS based on domains identified by various databases (list of domains shown in Figure S1A); any gene containing one or more of these domains was considered to be a chromatin-modifying factor. Using gene set enrichment analysis (GSEA) on human RMS microarray data sets, we found that this list of putative or known chromatin modifiers was significantly upregulated in human ERMS and ARMS (p<0.05, Figure S1B,C). We also searched for particularly families of chromatin factors to test. A total of nineteen human factors were chosen for analysis, including ten that represent two important classes of chromatin-modifying factors (SET or chromo domain proteins), and ten of the most highly upregulated genes in human ERMS versus normal muscle (Figure S1D; Figure S2). Of these factors, SUV39H1 emerged as the strongest modifier, significantly suppressing tumor formation from 12 dpf by the logrank test (p = 0.0001, Figure 1B).

### Suppression by SUV39H1 occurs early and is dependent on methyltransferase activity

**Figure 2:**
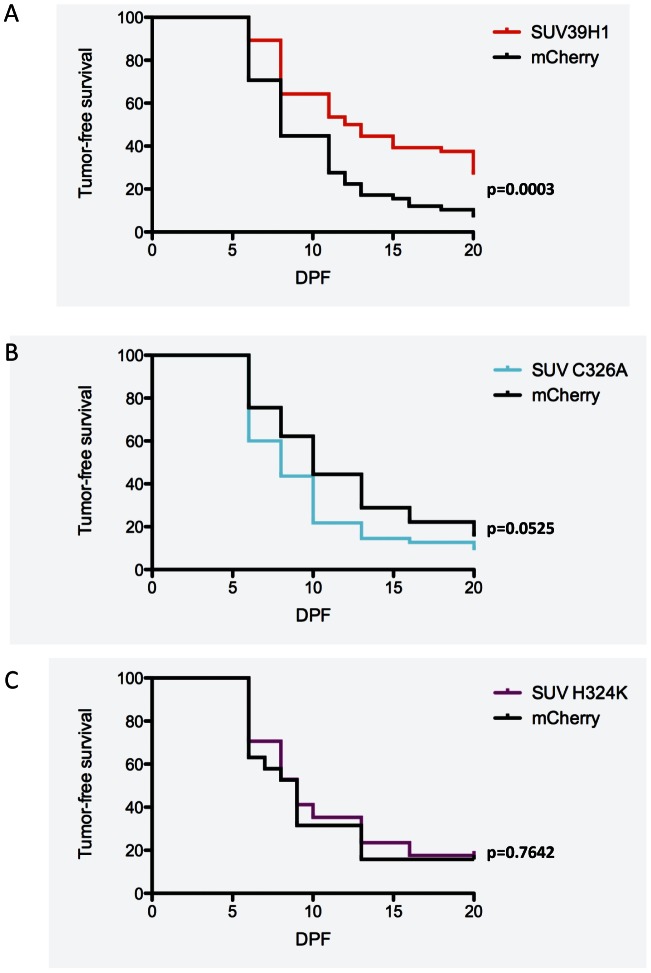
Suppression by SUV39H1 depends on enzymatic SET domain. (A) Injections into one-cell stage embryos of *rag2*-hKRAS^G12D^, *rag2*-hSUV39H1, *rag2*-GFP, and *mylz2*-mCherry, then selected for mCherry-high embryos, and monitored for tumor formation by GFP presence, results in a tumor-free survival curve that is significantly suppressed compared to the *rag2*-mCherry control curves. (SUV39H1 n = 56, mCherry n = 58, p = 0.0003). (B) Injection of the point mutant SUV39H1^C326A^ results in a tumor curve not significantly different from the mCherry control curve. However, this curve is significantly different from *rag2*-hSUV39H1 tumors (C326A n = 57, mCherry n = 48, p = 0.0525). (C) Similar injections of the *rag2*-hSUV39H1^H324K^ point mutant also results in a tumor curve like the *rag2*-mCherry curve and significantly different from the *rag2*-hSUV39H1 curve (H324K n = 17, mCherry n = 19, p = 0.7642).

SUV39H1-overexpressing tumors had a delay in tumor onset, so the Kaplan-Meier curve appears suppressed from the beginning of the assay at 12 dpf. To examine tumor formation with a more sensitive and quantitative assay, we used a quantitative fluorescent assay with a quadruple injection approach. As before, zebrafish were injected with *rag2*-hKRAS^G12D^ and either *rag2*-hSUV39H1 or, as a control, *rag2-*mCherry. The third construct was *rag2*-GFP to track the tumors by fluorescence as they arose, as it has been shown that 100% of *rag2*-GFP-positive foci go on to eventually form a tumor [23]. This enabled us to begin to see tumors days earlier than by the naked eye alone, thus resulting in shifted tumor curves relative to Figure 1B. The fourth construct was *mylz2*-mCherry to continue analyzing only successfully microinjected embryos, scored in 2 dpf embryos as for the screen. The inclusion of *rag2*-mCherry as a control did not interfere with the ability to score injections due to the timing difference in expression, since the *rag2* promoter turns on several days later. Because we track tumor formation with *rag2*-GFP, our control for *rag2*-SUV39H1, *rag2*-mCherry, does not confound our experimental results. By analyzing GFP fluorescence in the musculature of highly mCherry mosaic larvae, SUV39H1 still significantly suppressed RMS formation at 20 dpf (p = 0.0003, Figure 2A).

To determine if the histone methyltransferase (HMT) activity of SUV39H1 played a role in tumor suppression, we utilized SUV39H1 constructs with point mutations in the enzymatic SET domain that have been shown to lack methyltransferase activity [24]. We co-injected the SUV39H1 H324K and C326A mutants along with *rag2*-hKRAS^G12D^, *rag2*-GFP, and *mylz2-*mCherry and analyzed early tumor formation by fluorescence. Expression of either point mutant resulted in tumor-free survival curves similar to the control mCherry-overexpression curve rather than the suppressed SUV39H1-overexpression curve (C326A p = 0.0525, H324K p = 0.7642, Figure 2B,C). This result indicates that the tumor suppression by SUV39H1 depends on the HMT activity of the SET domain in SUV39H1, ruling out primarily scaffold effects since this enzyme is a part of a multiprotein complex.

### Characterization of SUV39H1-overexpressing tumors

**Figure 3:**
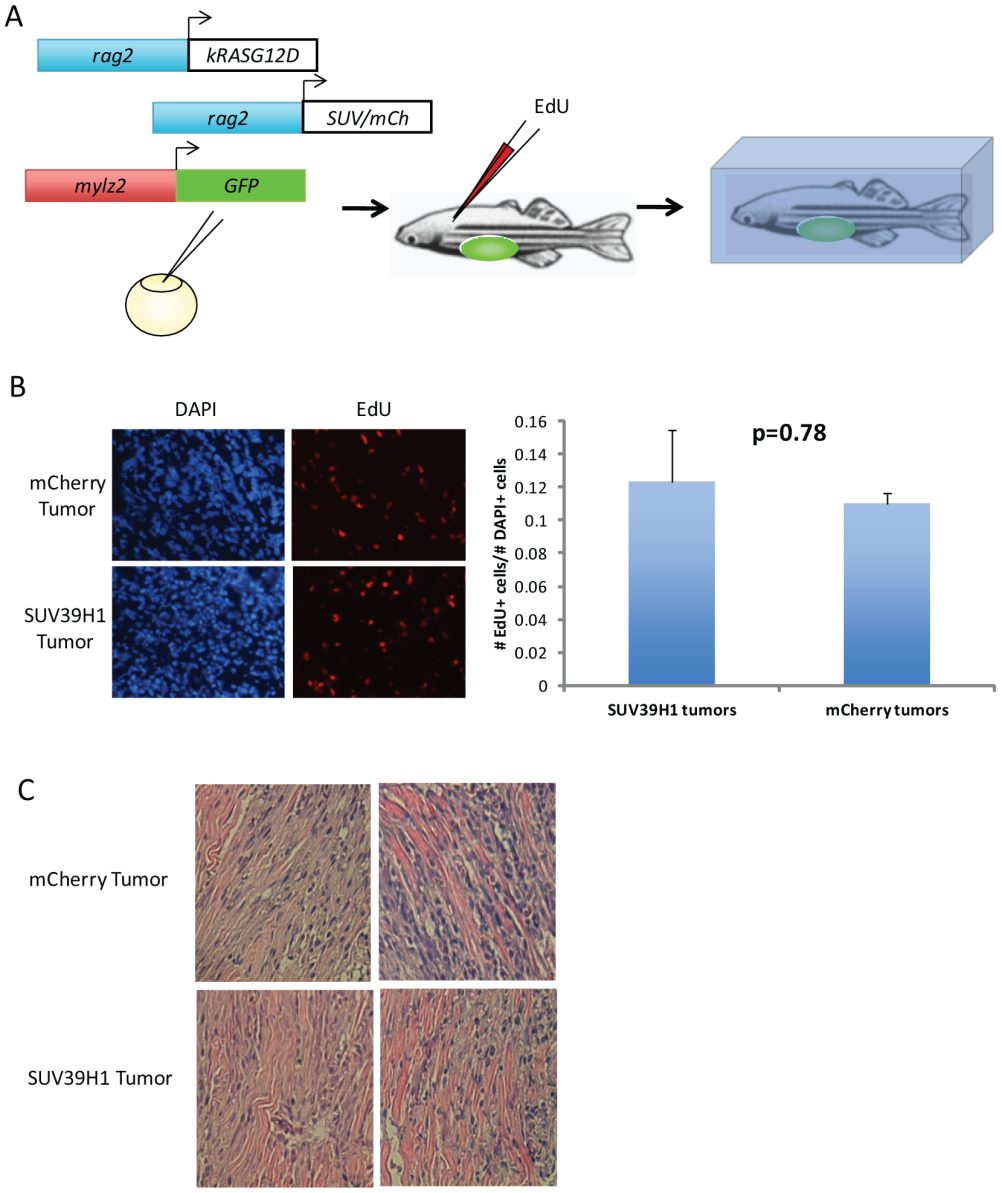
SUV39H1 overexpression does not impact cell cycle or muscle differentiation status of mature tumors. (A) Experimental design for *in vivo* cell cycle analysis. One-cell stage embryos were injected with three constructs *rag2*-hKRAS^G12D^, either *rag2*-mCherry or *rag2*-hSUV39H1, and *mylz2*-GFP. High-GFP expressing fish were raised to 30 dpf, and those with tumors were injected IP with EdU. After 24 hours, the injected fish were sacrificed and cryopreserved. (B) Number of EdU-positive cells, stained on GFP-positive tumor sections, was normalized to number of total cells, determined by presence of DAPI. No difference was observed between the control and SUV39H1 tumors (n = 5 for each group; p = 0.78). (C) H&E staining of RMS tumors overexpression either mCherry or SUV39H1. Both sets of tumors are very poorly differentiated; they also look similar to each other, indicating no difference in differentiation state of the tumors (bars represent 50 um, n = 6 for each group).

Since SUV39H1 is known to play a role in regulation of cell cycle and S phase genes [25]–[28], we investigated whether there was any difference in cell cycle rate between SUV39H1-overexpressing tumors and control tumors overexpressing mCherry. 5-ethynyl-2′-deoxyuridine (EdU), a bromodeoxyuridine analog, was injected intraperitoneally into five 30 dpf fish of each tumor type. Twenty-four hours post injection, the fish were sacrificed, embedded in OCT, and cryosectioned (Figure 3A). Staining for EdU in GFP-positive tumor sections revealed no difference in the percentage of cells dividing during labeling when normalized to cell number by DAPI staining, indicating no difference in cell cycle rate in the SUV39H1-overexpressing and control tumors (Student's t-test, p = 0.78, Figure 3B).

We wondered if muscle differentiation may be affected in the SUV39H1-expressing tumors, since SUV39H1 is known to regulate the master muscle regulator MyoD [29]. H&E staining on SUV39H1 and control tumors revealed no obvious changes in muscle differentiation status at the gross histologic level. Both sets of tumors were in an undifferentiated state, with higher cellularity and mostly mononucleated cells (Figure 3C). Global gene expression analysis of control and SUV39H1 tumors did not identify major differences (Figure S3). These studies demonstrate that overexpression of SUV39H1 did not significantly affect cell cycle rate or the differentiation state of *rag2*-hKRAS^G12D^ RMS tumors, at least at the gross histological level.

### SUV39H1 suppresses RMS tumor initiation

**Figure 4:**
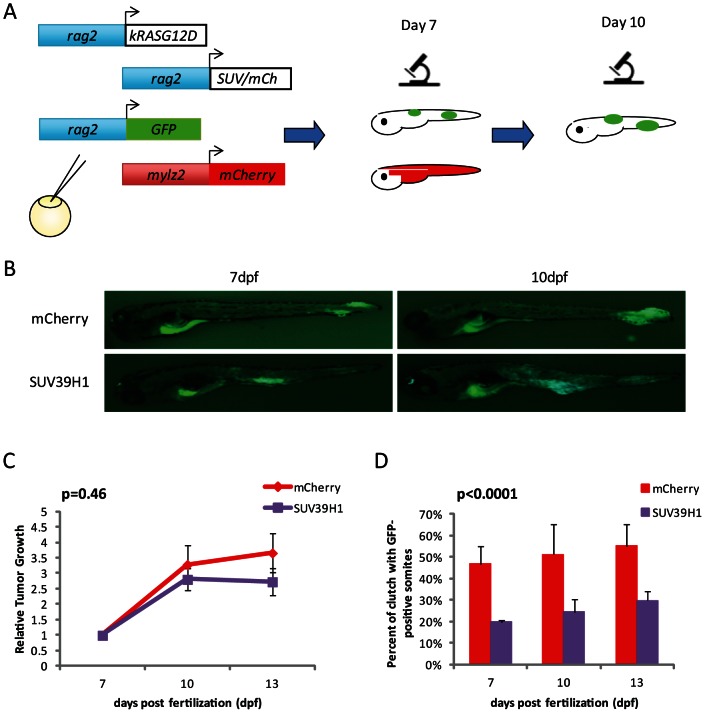
SUV39H1 impacts the initiation, not the growth rate, of the tumors. (A) Experimental design to view the tumors in larval stages by fluorescence. Four constructs are injected into one cell stage embryos, *rag2*-hKRAS^G12D^, *rag2-*hSUV39H1 or control *rag2*-mCherry, *rag2*-GFP, and *mylz2*-mCherry. Fish highly mosaicism for *mylz2*-mCherry expression are analyzed on day 7 for presence of GFP, indicative of developing tumors. Analysis is also performed on day 10 to note tumor growth. (B) Representative images of 7 and 10 dpf larvae with GFP-positive cells in the musculature. There is visible growth between 7 and 10 dpf as they develop into tumors. (C) Tumor growth rates, relative to size of tumor on day 7. There is no significant difference between growth rates of the SUV39H1-overexpressing tumors compared to control tumors (SUV39H1 n = 22, mCherry n = 13, p = 0.46). (D) Percentage of larvae within a clutch that contain GFP-positive cells in the musculature. More fish in the mCherry clutches have developing tumors when compared to SUV39H1 clutches (SUV39H1 n = 200, mCherry n = 136, p<0.0001).

The suppressive phenotype of SUV39H1 tumors in the Kaplan-Meier survival curves has already occurred at the first time point of 12 dpf (Figure 1C). To examine the earliest changes underlying this tumor suppression, we utilized the quadruple co-injection approach described above and shown in Figure 2. At 7 dpf, highly mCherry mosaic larval fish were examined for the presence of GFP in the musculature. Those that had GFP positive cells at 7 dpf were examined again at 10 and 13 dpf for the growth of these fluorescent patches into tumors (Figure 4A). In 100% of cases, the GFP-positive patches went on to produce a tumor, some even during this early time period examined (Figure 4B). The size of the developing tumors was quantified by measuring the number of fluorescent pixels in the image using the image analysis software ImageJ. Relative growth rates for SUV39H1 and control cohorts revealed that both sets of tumors grew at the same rate (Student's t-test, p = 0.46, Figure 4C). Within clutches containing equivalent levels of mCherry mosaicism, the number of larvae in a given clutch with GFP-positive patches was evaluated in the musculature. Significantly fewer patches were found in the SUV39H1-overexpression clutches compared to controls at 7 days (Fisher's exact test, p<0.0001, Figure 4D). This study in larvae demonstrated that SUV39H1 overexpression impacts the initiation of tumors. TUNEL analysis on 7 dpf larval sections revealed no differences in apoptotic levels between SUV39H1 and control larvae (p = 0.26, Student's t-test, Figure S4), eliminating increased cell death as the cause of reduced tumor initiation. Our data indicate that SUV39H1 overexpression affects the initiation of tumors, but once the tumor initiates, it grows at the same rate as control tumors.

**Figure 5:**
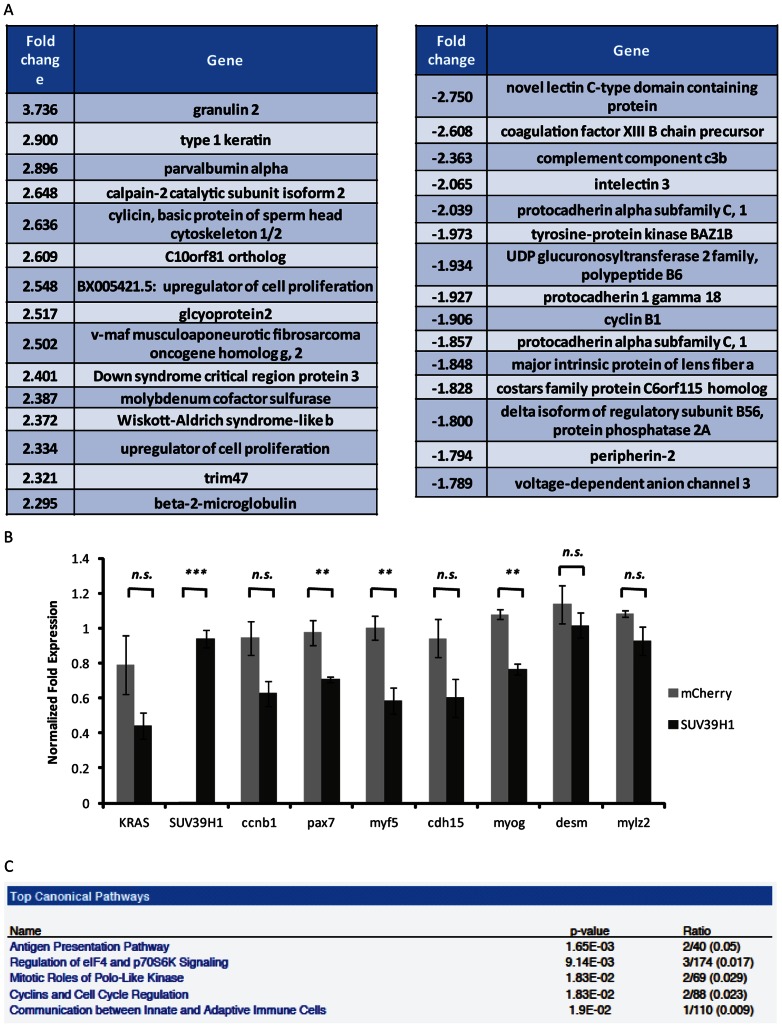
Global gene expression analysis reveals SUV39H1 may act through aberrant cell cycle regulation. (A) Table of top up- and downregulated annotated genes in *rag2*-KRAS^G12D^, *rag2*-SUV39H1 7 dpf larvae as compared to *rag2*-KRAS^G12D^, *rag2*-mCherry control larvae. Of particular interest was cyclin B1. (B) Gene expression analysis by qPCR in *rag2*-KRAS^G12D^, *rag2*-SUV39H1 7 dpf larvae as compared to *rag2*-KRAS^G12D^, *rag2*-mCherry control larvae, all *mylz2*-GFP positive. Cyclin B1 downregulation was approaching significance, confirming the microarray analysis (p = 0.0553). As expected, SUV39H1 levels differ between the larvae, but KRAS levels do not, suggesting SUV39H1 is not simply downregulating KRAS to suppress tumor formation (SUV39H1 p<0.0001; KRAS p = 0.1284). SUV39H1 may also have an impact on some muscle differentiation genes at this stage of tumor development (pax7 p = 0.0230; myf5 p = 0.0143; cdh15 p = 0.0922; myog p = 0.0017; desm p = 0.4098; mylz2 p = 0.1311). (C) Ingenuity Pathway Analysis of top canonical pathways affected based on the 7 dpf microarray list. Of the top five pathways impacted, two involve cell cycle regulators.

As Figure 4 demonstrates, there is already a significant difference between control and SUV39H1-overexpressing tumors at 7 dpf. However, *rag2*-GFP fluorescence is rarely visualized at 5 dpf, suggesting that the first tumors initiate between 5 and 7 dpf. To reveal what factors SUV39H1 could be repressing in the initiation of RMS, we performed global gene expression analysis on 5 and 7 dpf *rag2*-hKRAS^G12D^, *rag2*-hSUV39H1/mCherry embryos highly mosaic for *mylz2*-mCherry. As expected, the 5 dpf SUV39H1-overexpressing larvae revealed no differences compared to mCherry controls (data not shown). The 7 dpf larvae revealed a gene set differentially expressed between SUV39H1-overexpressing and control fish (top genes shown in Figure 5A). Of particular note is that cyclin B1 is downregulated in the SUV39H1-expressing fish (Figure 5A). Follow-up with 7 dpf larvae confirmed that cyclin B1 is indeed downregulated in SUV39H1-overexpressing larvae compared with control larvae, with the level of downregulation approaching significance (p = 0.0553, Figure 5B); additionally, this downregulation persists as the tumors mature, as tumors from 30 dpf SUV39H1-overexpressing fish demonstrated significantly lower levels of cyclin B1 compared with control fish (p = 0.0035, Figure S3). Similarly, Ingenuity Pathway Analysis of the top up- and down-regulated genes reveals that cyclins, polo-like kinase, and other cell cycle regulators are among the top canonical pathways differentially expressed in the SUV39H1-overexpressing fish (Figure 5C). This suggests that one role of SUV39H1 in the tumorigenic program is to suppress cell cycle entry.

## Discussion

Chromatin-modifying factors have increasingly been found to play an important role in tumorigenesis, particularly among pediatric rhabdomyosarcomas. Here, we describe an overexpression screen of chromatin-modifying factors that revealed SUV39H1 suppresses the onset of rhabdomyosarcoma formation in zebrafish. This effect is specific to the histone methyltransferase activity of SUV39H1, as point mutations in the SET domain lacked the suppressive effect. We have demonstrated that the effects are not simply due to SUV39H1 directly suppressing KRAS^G12D^ expression, as oncogenic KRAS mRNA levels do not significantly differ between SUV39H1-overexpressing and control larvae and tumors. While the SUV39H1-overexpressing mature tumors do not display differences in tumor proliferation and muscle differentiation status, our studies establish that larval fish with ectopic SUV39H1 expression initiate fewer tumors.

The requirement for the wild-type SET domain in our model is striking since this was not the case of HMTs in a zebrafish melanoma model. When SETDB1 was found to accelerate melanoma, two methyltransferase-deficient SETDB1 mutants also had an accelerated tumor incidence curve, likely because the complex still had methyltransferase activity [20]. This suggests that RMS formation is more sensitive than melanoma formation to the loss of SUV39H1 methyltransferase activity. It also suggests that the suppressive phenotype of wild-type SUV39H1 overexpression is not caused by a scaffold or dominant negative effect.

Global gene expression analysis comparing *rag2*-hKRAS^G12D^/*rag2*-hSUV39H1 with *rag2*-hKRAS^G12D^/*rag2*-mCherry 7dpf larvae revealed that cyclin B1 is downregulated in the SUV39H1-overexpressing tumors, which was confirmed by qPCR. SUV39H1 is already known to silence cyclins E and A [30]. Silencing of cyclin B1 may explain how SUV39H1 promotes senescence and growth arrest. Quiescence can often be mediated by cyclin B1 downregulation, as in CD34-negative hematopoietic stem cells and quiescent NIH3T3 cells [31], [32]. Sp1, a cell growth and survival transcription factor, has been shown to associate with SUV39H1 upon hydrogen peroxide treatment in an epithelial carcinoma cell line, leading to growth arrest through the silencing of Sp1 target genes, including cyclin B1 [33]. SUV39H1 overexpression may silence cyclin B1, leading to growth arrest and decreased tumor initiation.

Previous studies involving SUV39H1 have demonstrated its role as a tumor suppressor involving cell cycle regulation. SUV39H1 has been shown to impact the cell cycle through regulation of senescence. SUV39H1 was demonstrated to associate with pRb as well as HDAC1, and a senescence response in melanocytes that acts through these two proteins is mediated by SUV39H1 heterochromatization [30], [34], [35]. In a murine model of Ras-driven T cell lymphoma, SUV39H1-dependent senescent growth arrest prevents the onset of tumorigenesis; this senescence is likely dependent on H3K9 methylation on specific growth genes [36]. Additionally, loss of SUV39H1 in Rb heterozygote mice leads to the development of C cell adenocarcinomas, along with frequent expression of proliferation markers, suggesting that SUV39H1 suppresses tumors through senescence [37]. When oncogenic NRAS was transduced into cell lines derived from Rb deficient tumors, senescence was induced and SUV39H1 was recruited to chromatin [37]. Loss of SUV39H1 in a myc-driven model of murine B cell lymphomas led to faster onset of disease, whereas SUV39H1 wild-type mice displayed increased levels of senescence and growth arrest [38]. It has been suggested that the SUV39H1-mediated H3K9me3 mark on heterochromatin is a widespread mark of the senescence program and that this program could be targeted for cancer therapies [38].

As a cell cycle regulator, SUV39H1 is also known to silence S phase genes as well as p21, both of which will induce growth arrest [25]–[28], [39]. SUV39H1 has been shown to associate with pRb in the context of keeping E2F and its cell cycle-promoting target genes are repressed through heterochromatization. SUV39H1 is phosphorylated at the G1/S transition to reduce its activity and allow for cell cycle promotion; when overexpressed, it has been shown to suppress cell growth. The identification of SUV39H1 as a suppressor of RMS onset in our zebrafish model supports these studies and the notion that SUV39H1 functions as a tumor suppressor [36], [40].

With SUV39H1 as a potential tumor suppressor in RMS, we looked to the Oncomine database to evaluate the status of SUV39H1 expression in human RMS. According to the Khan Multi-cancer study, SUV39H1 expression is down in RMS, including both ERMS and ARMS subtypes, compared to skeletal muscle tissue samples. This is consistent with our study, where overexpression leads to reduced tumor formation. Therefore, future research should investigate the pathways that SUV39H1 regulates in its role as a tumor suppressor, with particular focus on cell cycle regulation. It also suggests that SUV39H1 may represent a putative therapeutic target, whereby increasing SUV39H1 expression may block cell cycle progression and halt tumor formation.

In conclusion, we performed a screen of chromatin-modifying factors for their effects on tumorigenesis in RMS using zebrafish as a model organism. Our screen revealed that chromatin-modifying factors do play a role in RMS formation. SUV39H1 was determined to be a suppressor of RMS formation, dependent on its histone methyltransferase activity, suppressing tumor initiation likely regulation of the cell cycle.

## Supporting Information

Figure S1 **Human RMS samples contain upregulation of chromatin-modifying factors.** (A) List of protein domains resulting in a list of chromatin-modifying factors. (B) Gene set enrichment analysis results show significant enrichment for chromatin factor gene lists in human embryonal RMS versus normal human juvenile muscle. (C) Gene set enrichment analysis results show significant enrichment for chromatin factor gene lists in human alveolar RMS versus normal human juvenile muscle (p<0.05 for B,C). (D) List of twenty chromatin-modifying factors screened for effects on RMS formation.
(TIF)

Figure S2 **Screen of twenty chromatin-modifying factors for their role in rhabdomyosarcoma formation.** Twenty chromatin-modifying factors were analyzed for their effects on RMS formation. Most did not result in significant differences from the four historical control curves (dsRed).
(TIF)

Figure S3 **SUV39H1 overexpression leads to downregulation of cyclin B1 expression in mature tumors but does not affect markers of muscle differentiation.** Gene expression analysis of tumors from *rag2*-hKRAS^G12D^, *rag2*-mCherry, and *mylz2*-mCherry-positive 30 dpf fish and *rag2*-hKRAS^G12D^, *rag2*-SUV39H1, and *mylz2*-mCherry-positive 30 dpf fish. The only gene tested with a significant difference between SUV39H1-overexpressing and control tumors was cyclin B1 (ccnb1, p = 0.0035), though SUV39H1 was nearly significant, as expected (p = 0.0533). The remaining genes had no differences between SUV39H1-overexpressing and control tumors, suggesting that neither KRAS levels nor muscle differentiation were the cause of tumor suppression (KRAS p = 0.5855; pax7 p = 0.5502; myf5 p = 0.1103; cdh15 p = 0.7720; myog p = 0.8831; desm p = 0.8739; mylz2 p = 0.2425).
(TIF)

Figure S4 **SUV39H1 overexpression does not lead to increased apoptosis.** (A) TUNEL staining of *rag2*-hKRAS^G12D^, *rag2*-mCherry, and *mylz2*-mCherry-positive 7 dpf fish (20×). (B) Similar levels of cell death are seen in *rag2*-hKRAS^G12D^, *rag2*-hSUV39H1, and *mylz2*-mCherry-positive 7 dpf fish, as noted by TUNEL staining (20×). (C) Average number of TUNEL-positive cells over two separate fields of musculature per larvae (n = 5 for each). This result reveals that there is not increased apoptosis in the SUV39H1-overexpressing larvae, suggesting the tumor initiating cells are not simply dying off (p = 0.26).
(TIF)

Table S1 **List of primer sequences used for quantitative RT-PCR.**
(DOCX)

## References

[1] Arndt C, Crist W (1999) Common musculoskeletal tumors of childhood and adolescence. N Engl J Med 341: 342–352. 10423470 10.1056/NEJM199907293410507

[2] Ruymann F, Grovas A (2000) Progress in the diagnosis and treatment of rhabdomyosarcoma and related soft tissue sarcomas. Cancer Invest 18: 223–241. 10754991 10.3109/07357900009031827

[3] Breitfeld P, Meyer W (2005) Rhabdomyosarcoma: new windows of opportunity. Oncologist 10: 518–527. 16079319 10.1634/theoncologist.10-7-518

[4] O'Brien D, Jacob A, Qualman S, Chandler D (2012) Advances in pediatric rhabdomyosarcoma characterization and disease model development. Histol Histopathol 27: 13–22. 22127592 10.14670/hh-27.13 PMC3922709

[5] Langenau D, Keefe M, Storer N, Guyon J, Kutok J, et al (2007) Effects of RAS on the genesis of embryonal rhabdomyosarcoma. Genes Dev 21: 1382–1395. 17510286 10.1101/gad.1545007 PMC1877750

[6] Stratton M, Fisher C, Gusterson B, Cooper C (1989) Detection of point mutations in N-ras and K-ras genes of human embryonal rhabdomyosarcomas using oligonucleotide probes and the polymerase chain reaction. Cancer Res 49: 6324–6327. 2680062

[7] Schwienbacher C, Sabbioni S (1998) Transcriptional map of 170-kb region at chromosome 11p15. 5: identification and mutational analysis of the BWR1A gene reveals the presence of mutations in tumor. Proc Natl Acad Sci U S A 95: 3873–3878. 9520460 10.1073/pnas.95.7.3873 PMC19930

[8] Xia S, Pressey J, Barr F (2002) Molecular pathogenesis of rhabdomyosarcoma. Cancer Biol Ther 1: 97–104. 12170781 10.4161/cbt.51

[9] Chen Y, Takita J, Hiwatari M, Igarashi T, Hanada R, et al (2006) Mutations of the PTPN11 and RAS genes in rhabdomyosarcoma and pediatric hematological malignancies. Genes Chromosomes Cancer 45: 583–591. 16518851 10.1002/gcc.20322

[10] Martinelli S, McDowell H, Vigne S, Kokai G, Uccini S, et al (2009) RAS signaling dysregulation in human embryonal Rhabdomyosarcoma. Genes Chromosomes Cancer 48: 975–982. 19681119 10.1002/gcc.20702

[11] Qualman S, Coffin C, Newton W, Hojo H (1998) Intergroup Rhabdomyosarcoma Study: update for pathologists. Pediatr Dev Pathol 1: 550–561. 9724344 10.1007/s100249900076

[12] Wang H, Garzon R, Sun H, Ladner K, Singh R, et al (2008) NF-kappaB-YY1-miR-29 regulatory circuitry in skeletal myogenesis and rhabdomyosarcoma. Cancer Cell 14: 369–381. 18977326 10.1016/j.ccr.2008.10.006 PMC3829205

[13] DeCristofaro M, Betz B, Wang W, Weissman B (1999) Alteration of hSNF5/INI1/BAF47 detected in rhabdoid cell lines and primary rhabdomyosarcomas but not Wilms' tumors. Oncogene 18: 7559–7565. 10602515 10.1038/sj.onc.1203168

[14] Uno K, Takita J, Yokomori K, Tanaka Y, Ohta S, et al (2002) Aberrations of the hSNF5/INI1 gene are restricted to malignant rhabdoid tumors or atypical teratoid/rhabdoid tumors in pediatric solid tumors. Genes Chromosomes Cancer 34: 33–41. 11921280 10.1002/gcc.10052

[15] Li Z-Y, Yang J, Gao X, Lu J-Y, Zhang Y, et al (2007) Sequential recruitment of PCAF and BRG1 contributes to myogenin activation in 12-O-tetradecanoylphorbol-13-acetate-induced early differentiation of rhabdomyosarcoma-derived cells. J Biol Chem 282: 18872–18878. 17468105 10.1074/jbc.M609448200

[16] Schildhaus H-U, Riegel R, Hartmann W, Steiner S, Wardelmann E, et al (2011) Lysine-specific demethylase 1 is highly expressed in solitary fibrous tumors, synovial sarcomas, rhabdomyosarcomas, desmoplastic small round cell tumors, and malignant peripheral nerve sheath tumors. Human Pathology 42: 1667–75. 21531005 10.1016/j.humpath.2010.12.025

[17] Bennani-Baiti I, Machado I, Llombart-Bosch A, Kovar H (2012) Lysine-specific demethylase 1 (LSD1/KDM1A/AOF2/BHC110) is expressed and is an epigenetic drug target in chondrosarcoma, Ewing's sarcoma, osteosarcoma, and rhabdomyosarcoma. Hum Pathol 10.1016/j.humpath.2011.10.010 22245111

[18] Westerfield M (1993) The zebrafish book: a guide for the laboratory use of zebrafish (Brachydanio rerio). Eugene: University of Oregon Press.

[19] Smith A, Raimondi A, Salthouse C, Ignatius M, Blackburn J, et al (2010) High-throughput cell transplantation establishes that tumor-initiating cells are abundant in zebrafish T-cell acute lymphoblastic leukemia. Blood 115: 3296–3303. 20056790 10.1182/blood-2009-10-246488 PMC2858492

[20] Ceol C, Houvras Y, Jane-Valbuena J, Bilodeau S, Orlando D, et al (2011) The histone methyltransferase SETDB1 is recurrently amplified in melanoma and accelerates its onset. Nature 471: 513–517. 21430779 10.1038/nature09806 PMC3348545

[21] Wachtel M, Dettling M, Koscielniak E, Stegmaier S, Treuner J, et al (2004) Gene expression signatures identify rhabdomyosarcoma subtypes and detect a novel t(2;2)(q35;p23) translocation fusing PAX3 to NCOA1. Cancer Res 64: 5539–5545. 15313887 10.1158/0008-5472.CAN-04-0844

[22] Langenau D, Keefe M, Storer N, Jette C, Smith A, et al (2008) Co-injection strategies to modify radiation sensitivity and tumor initiation in transgenic Zebrafish. Oncogene 27: 4242–4248. 18345029 10.1038/onc.2008.56 PMC2680704

[23] Le X, Pugach EK, Hettmer S, Storer NY, Liu J, et al (2013) A novel chemical screening strategy in zebrafish identifies common pathways in embryogenesis and rhabdomyosarcoma development. Development 140: 2354–2364. 23615277 10.1242/dev.088427 PMC3653557

[24] Rea S, Eisenhaber F, O'Carroll D, Strahl B, Sun Z, et al (2000) Regulation of chromatin structure by site-specific histone H3 methyltransferases. Nature 406: 593–599. 10949293 10.1038/35020506

[25] Vandel L, Nicolas E, Vaute O, Ferreira R, Ait-Si-Ali S, et al (2001) Transcriptional repression by the retinoblastoma protein through the recruitment of a histone methyltransferase. Mol Cell Biol 21: 6484–6494. 11533237 10.1128/MCB.21.19.6484-6494.2001 PMC99795

[26] Ait-Si-Ali S, Guasconi V, Fritsch L, Yahi H, Sekhri R, et al (2004) A Suv39h-dependent mechanism for silencing S-phase genes in differentiating but not in cycling cells. EMBO J 23: 605–615. 14765126 10.1038/sj.emboj.7600074 PMC1271807

[27] Giacinti C, Giordano A (2006) RB and cell cycle progression. Oncogene 25: 5220–5227. 16936740 10.1038/sj.onc.1209615

[28] Firestein R, Cui X, Huie P, Cleary M (2000) Set domain-dependent regulation of transcriptional silencing and growth control by SUV39H1, a mammalian ortholog of Drosophila Su(var)3-9. Mol Cell Biol 20: 4900–4909. 10848615 10.1128/mcb.20.13.4900-4909.2000 PMC85941

[29] Mal A (2006) Histone methyltransferase Suv39h1 represses MyoD-stimulated myogenic differentiation. EMBO J 25: 3323–3334. 16858404 10.1038/sj.emboj.7601229 PMC1523181

[30] Nielsen S, Schneider R, Bauer U, Bannister A, Morrison A, et al (2001) Rb targets histone H3 methylation and HP1 to promoters. Nature 412: 561–565. 11484059 10.1038/35087620

[31] Huss R, Gatsios P, Graeve L, Lange C, Eissner G, et al (2000) Quiescence of CD34-negative haematopoietic stem cells is mediated by downregulation of Cyclin B and no stat activation. Cytokine 12: 1195–1204. 10930296 10.1006/cyto.1999.0732

[32] Farina A, Gaetano C, Crescenzi M, Puccini F, Manni I, et al (1996) The inhibition of cyclin B1 gene transcription in quiescent NIH3T3 cells is mediated by an E-box. Oncogene 13: 1287–1296. 8808703

[33] Chuang J-Y, Chang W-C, Hung J-J (2011) Hydrogen peroxide induces Sp1 methylation and thereby suppresses cyclin B1 via recruitment of Suv39H1 and HDAC1 in cancer cells. Free Radic Biol Med 51: 2309–2318. 22036763 10.1016/j.freeradbiomed.2011.10.001

[34] Vaute O, Nicolas E, Vandel L, Trouche D (2002) Functional and physical interaction between the histone methyl transferase Suv39H1 and histone deacetylases. Nucleic Acids Res 30: 475–481. 11788710 10.1093/nar/30.2.475 PMC99834

[35] Bandyopadhyay D, Curry J, Lin Q, Richards H, Chen D, et al (2007) Dynamic assembly of chromatin complexes during cellular senescence: implications for the growth arrest of human melanocytic nevi. Aging Cell 6: 577–591. 17578512 10.1111/j.1474-9726.2007.00308.x PMC1974778

[36] Braig M, Lee S, Loddenkemper C, Rudolph C, Peters A, et al (2005) Oncogene-induced senescence as an initial barrier in lymphoma development. Nature 436: 660–665. 16079837 10.1038/nature03841

[37] Shamma A, Takegami Y, Miki T, Kitajima S, Noda M, et al (2009) Rb Regulates DNA damage response and cellular senescence through E2F-dependent suppression of N-ras isoprenylation. Cancer Cell 15: 255–269. 19345325 10.1016/j.ccr.2009.03.001

[38] Reimann M, Lee S, Loddenkemper C, Dörr J, Tabor V, et al (2010) Tumor stroma-derived TGF-beta limits myc-driven lymphomagenesis via Suv39h1-dependent senescence. Cancer Cell 17: 262–272. 20227040 10.1016/j.ccr.2009.12.043

[39] Cherrier T, Suzanne S, Redel L, Calao M, Marban C, et al (2009) p21(WAF1) gene promoter is epigenetically silenced by CTIP2 and SUV39H1. Oncogene 28: 3380–3389. 19581932 10.1038/onc.2009.193 PMC3438893

[40] Peters A, O'Carroll D, Scherthan H, Mechtler K, Sauer S, et al (2001) Loss of the Suv39h histone methyltransferases impairs mammalian heterochromatin and genome stability. Cell 107: 323–337. 11701123 10.1016/s0092-8674(01)00542-6

