# Supplementary figures and images for: The Histone Methyltransferase SUV39H1 Suppresses Embryonal Rhabdomyosarcoma Formation in Zebrafish
Source: PLoS One. 2013 May 21;8(5):e64969. doi: 10.1371/journal.pone.0064969 (PMC3660348; doi:10.1371/journal.pone.0064969)

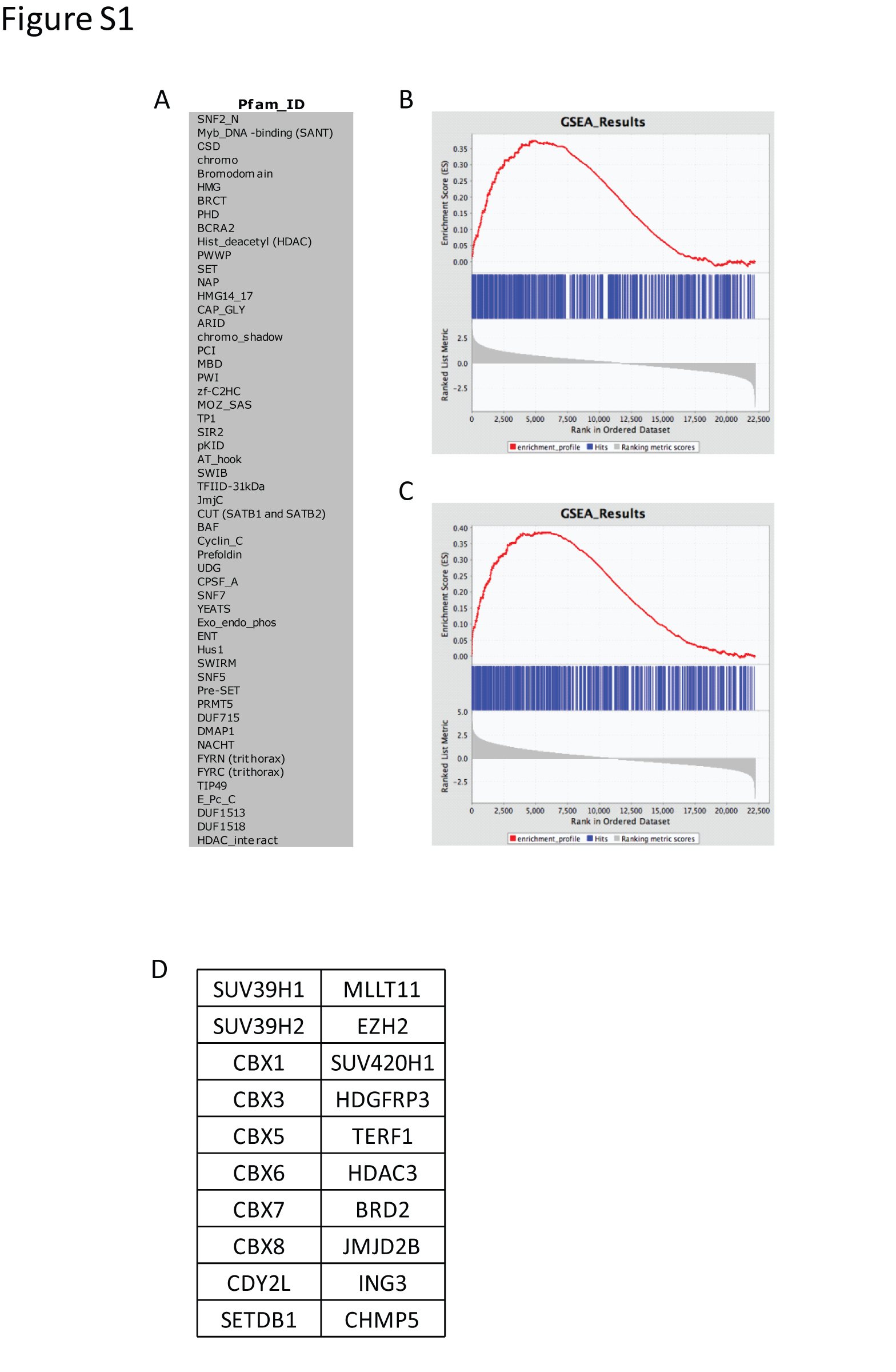

Supplement: Figure S1 — Human RMS samples contain upregulation of chromatin-modifying factors. (A) List of protein domains resulting in a list of chromatin-modifying factors. (B) Gene set enrichment analysis results show significant enrichment for chromatin factor gene lists in human embryonal RMS versus normal human juvenile muscle. (C) Gene set enrichment analysis results show significant enrichment for chromatin factor gene lists in human alveolar RMS versus normal human juvenile muscle (p<0.05 for B,C). (D) List of twenty chromatin-modifying factors screened for effects on RMS formation. (TIF) [file pone.0064969.s001.tif]

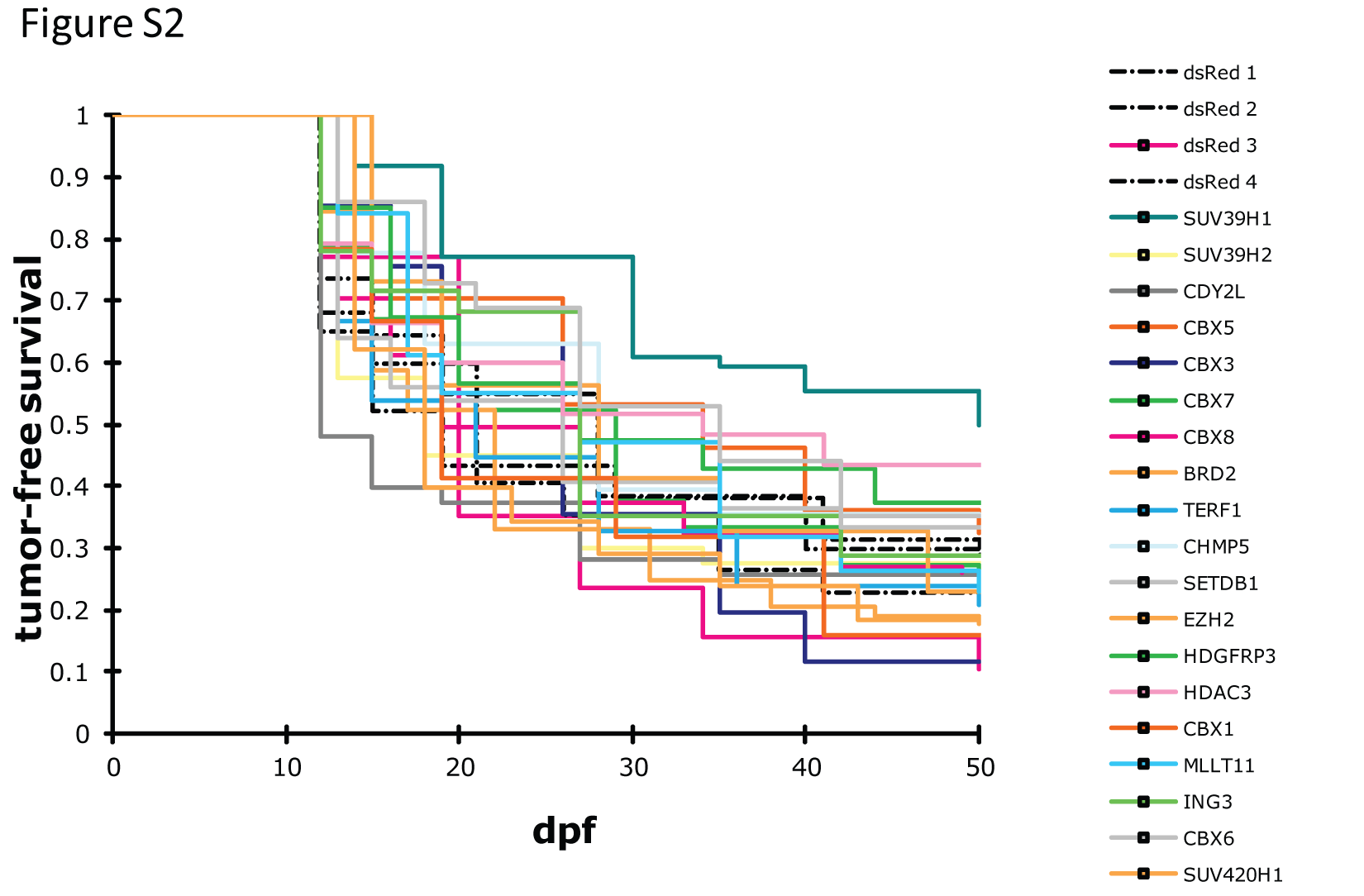

Supplement: Figure S2 — Screen of twenty chromatin-modifying factors for their role in rhabdomyosarcoma formation. Twenty chromatin-modifying factors were analyzed for their effects on RMS formation. Most did not result in significant differences from the four historical control curves (dsRed). (TIF) [file pone.0064969.s002.tif]

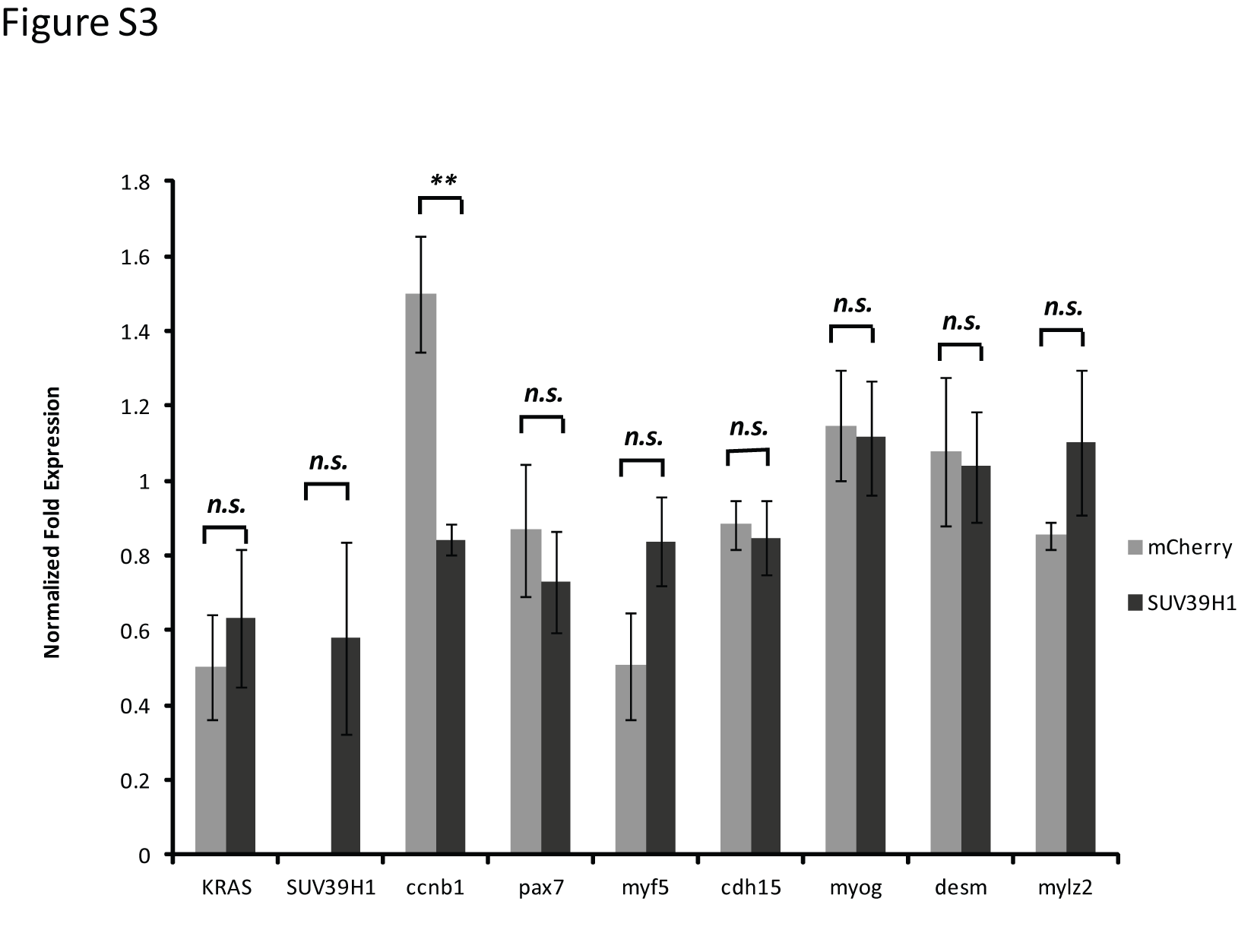

Supplement: Figure S3 — SUV39H1 overexpression leads to downregulation of cyclin B1 expression in mature tumors but does not affect markers of muscle differentiation. Gene expression analysis of tumors from rag2-hKRASG12D, rag2-mCherry, and mylz2-mCherry-positive 30 dpf fish and rag2-hKRASG12D, rag2-SUV39H1, and mylz2-mCherry-positive 30 dpf fish. The only gene tested with a significant difference between SUV39H1-overexpressing and control tumors was cyclin B1 (ccnb1, p = 0.0035), though SUV39H1 was nearly significant, as expected (p = 0.0533). The remaining genes had no differences between SUV39H1-overexpressing and control tumors, suggesting that neither KRAS levels nor muscle differentiation were the cause of tumor suppression (KRAS p = 0.5855; pax7 p = 0.5502; myf5 p = 0.1103; cdh15 p = 0.7720; myog p = 0.8831; desm p = 0.8739; mylz2 p = 0.2425). (TIF) [file pone.0064969.s003.tif]

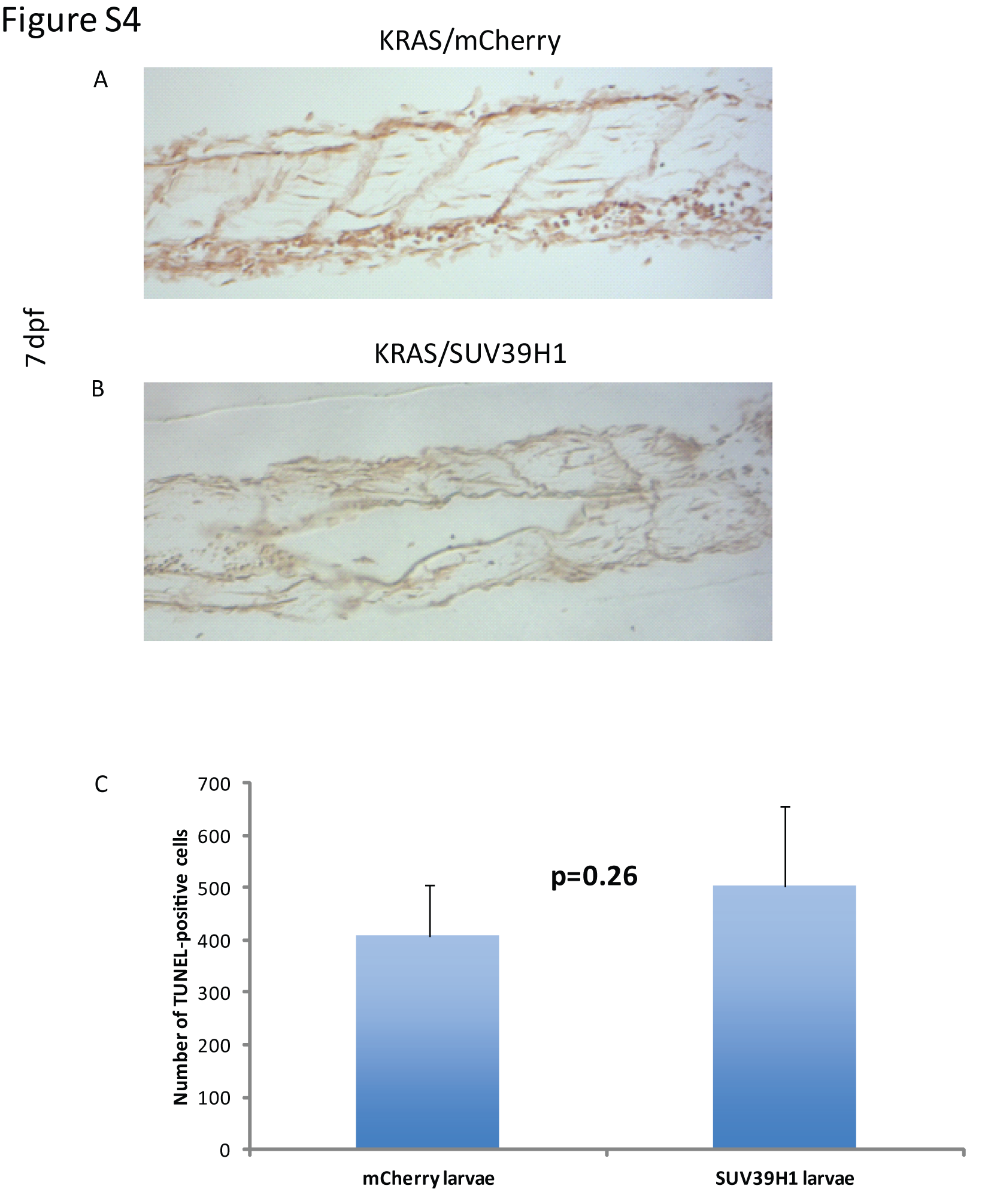

Supplement: Figure S4 — SUV39H1 overexpression does not lead to increased apoptosis. (A) TUNEL staining of rag2-hKRASG12D, rag2-mCherry, and mylz2-mCherry-positive 7 dpf fish (20×). (B) Similar levels of cell death are seen in rag2-hKRASG12D, rag2-hSUV39H1, and mylz2-mCherry-positive 7 dpf fish, as noted by TUNEL staining (20×). (C) Average number of TUNEL-positive cells over two separate fields of musculature per larvae (n = 5 for each). This result reveals that there is not increased apoptosis in the SUV39H1-overexpressing larvae, suggesting the tumor initiating cells are not simply dying off (p = 0.26). (TIF) [file pone.0064969.s004.tif]
