# Supplementary material for: The Histone Methyltransferase SUV39H1 Suppresses Embryonal Rhabdomyosarcoma Formation in Zebrafish
Source: PLoS One. 2013 May 21;8(5):e64969. doi: 10.1371/journal.pone.0064969 (PMC3660348; doi:10.1371/journal.pone.0064969)
Supplement: Table S1 — List of primer sequences used for quantitative RT-PCR. (DOCX) [file pone.0064969.s005.docx]

**Table S1**

| **Primers for quantitative RT-PCR** | |
| --- | --- |
| EF1a-QRT-F | 5’-CATCGAGAAGTTCGAGAAGGAAGC-3’ |
| EF1a-QRT-R | 5’-GTCAATGGTGATACCACGCTCAC-3’ |
| hKRAS-QRT-F | 5’-TTGATGGAGAAACCTGTCTCTTGG-3’ |
| hKRAS-QRT-R | 5’-CAAATACACAAAGAAAGCCCTCCC-3’ |
| pax7b-QRT-F | 5’-CAGTATTGACGGCATTCTGGGAG-3’ |
| pax7b-QRT-R | 5’-TCTCTGCTTTCTCTTGAGCGGC-3’ |
| myf5-QRT-F | 5’-CCAGACAGTCCAAACAACAGACC-3’ |
| myf5-QRT-R | 5’-TGAGCAAGCAGTGTGAGTAAGCG-3’ |
| cdh15-QRT-F | 5'-CTAAGGAAAGATGCACCCCATTAC-3’ |
| cdh15-QRT-R | 5’-TCAGAGCTGTGTCGTATGGTGG-3’ |
| myog-QRT-F | 5’-GTGGACAGCATAACGGGAACAG-3’ |
| myog-QRT-R | 5’-TCTGAAGGTAACGGTGAGTCGG-3’ |
| desmin-QRT-F | 5’-CGAGATTGACTCTCTCAAGGGCAC-3’ |
| desmin-QRT-R | 5’-GGGCGATAGTGTCCTGATAACCAC-3’ |
| mylz2-QRT-F | 5’-TTGACCACTCAGTGCGACAGGTTC-3’ |
| mylz2-QRT-R | 5’-AACATTGCCAGCCACATCTGGG-3’ |
| ccnb1-QRT-F | 5’-GGACTCAGACCAAGGGCCGC-3’ |
| ccnb1-QRT-R | 5’-GCACAGCCGGAGGTCTCCAT-3’ |
